# Reduction in drinking days and binge drinking days among patients receiving screening, brief intervention, and referral to treatment services during an emergency department visit: six-month results

**DOI:** 10.1186/1940-0640-7-S1-A97

**Published:** 2012-10-09

**Authors:** Joanna Akin, Aaron Johnson, J Paul Seale, Gabe Kuperminc

**Affiliations:** 1 Department of Psychology, Georgia State University, Decatur, GA, USA; 2 Department of Medicine, Mercer University School of Medicine, Macon, GA, USA; 3 Department of Family Medicine, Mercer University School of Medicine, Macon, GA, USA; 4 Department of Psychology, Georgia State University, Atlanta, GA, USA

## 

Alcohol screening and brief intervention (SBI) is effective in many health-care settings. Previous research has shown significant decreases in harmful drinking due to SBI, but many studies, particularly in emergency/trauma settings, did not use a control group. Thus, it is unclear if observed decreases in harmful drinking are due to the intervention or other factors such as the hospital visit, the substance use assessment, or simply regression to the mean. This project assessed the effectiveness of an SBI program implemented at an urban hospital in the US state of Georgia. Participants were enrolled between January 2009 and April 2010 during an emergency department visit. Intervention patients (n = 120) and control patients (n = 400) were assessed using the Alcohol, Smoking, and Substance Involvement Screening Test (ASSIST) and completed a survey that measured harmful drinking, among other items. Intervention patients received a 10-15 minute motivational-interviewing–based brief intervention aimed at reducing harmful drinking. All patients were contacted six months later for follow-up assessment. Analyses investigated between-group differences in alcohol consumption over time. Among intervention group patients, results show that past 30-day mean drinking days were reduced from 11.7 days at baseline to 5.8 days at six months and mean binge drinking days were reduced from 7.6 days at baseline to 3.0 days at six months. Although the control group also showed significant reductions in mean drinking days and heavy episodic drinking days over time, regression analysis indicated a significantly greater reduction in heavy episodic drinking days for the intervention group. Further analyses examined the impact of the intervention across different risk levels, based on ASSIST score. Preliminary results suggest those with moderate-risk drinking received the most benefit from the brief intervention. These results provide further evidence of SBI’s effectiveness in reducing harmful drinking.

